# Supplementary material for: Dopamine Receptor Activation Modulates the Integrity of the Perisynaptic Extracellular Matrix at Excitatory Synapses
Source: Cells. 2020 Jan 21;9(2):260. doi: 10.3390/cells9020260 (PMC7073179; doi:10.3390/cells9020260)
Supplement: Supplementary file 1 [file cells-09-00260-s001.pdf]

Supplementary Figure S1: D1Rs are expressed in rat dissociated cortical neurons and their stimulation leads to increased synaptic activity

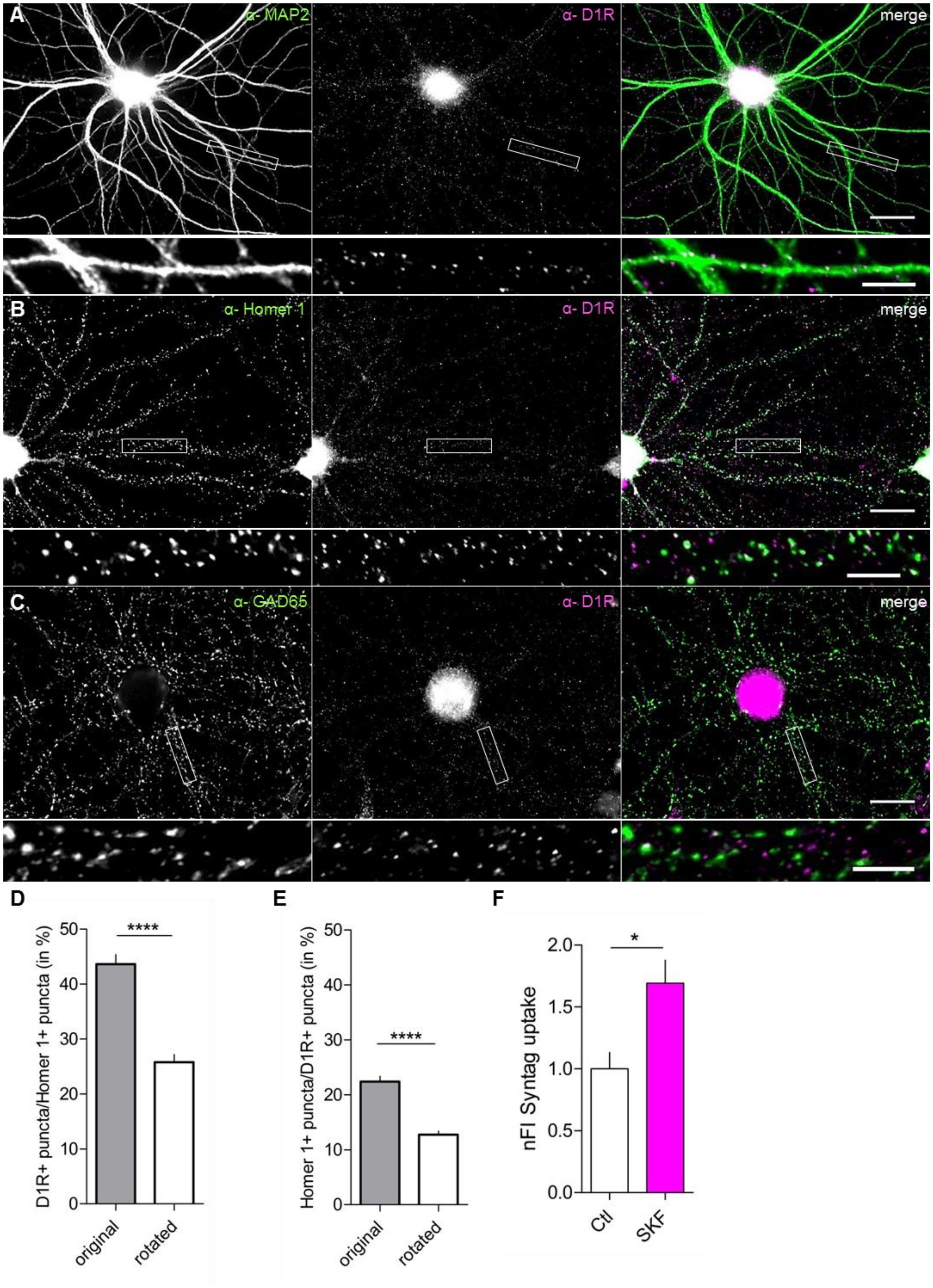

A, B, C) Rat dissociated cortical neurons (DIV21) were stained for the somato-dendritic marker MAP2 (A, green), the excitatory synaptic marker Homer 1 (B, green) or the inhibitory synaptic marker GAD65 (C, green) and D1Rs (magenta). D1Rs appear as bright, little puncta along dendrites (A) and many of them in close vicinity of Homer 1-positive synaptic sites (B). D1R-positive puncta also appear in close vicinity of GAD65-positive synaptic puncta (C) (scale bar: 20 µm; close-up: 5 µm). **D)** In rat dissociated cortical cultures (DIV21) around 44 % of D1R-positive puncta are in close vicinity of Homer 1-positive synaptic puncta. The analysis of a 90° rotated image served as a quality control (original, 43.65 ± 1.727, n = 6; rotated, 25.79 ± 1.389, n = 6; average % ± SEM %; Paired t test; **** P < 0.0001). **E)** Around 22 % of Homer 1-positive synaptic puncta can be found in close vicinity of D1R-positive puncta. The analysis of a 90° rotated image served as a quality control (original, 22.41 ± 0.9848, n = 6; rotated, 12.75 ± 0.6574, n = 6; average % ± SEM %; Paired t test; **** P < 0.0001). **F)** Presynaptic activity is enhanced after stimulation of D1-like DA receptors by SKF81297 (Ctl, 1± 0.1326, n = 6; SKF, 1,692 ± 0.1849, n = 6; average ± SEM; Unpaired t test; * P = 0.0124) (nFI Syntag uptake = normalized fluorescent intensity of synaptotagmin uptake).

Supplementary Figure S2: D1R-positive puncta can be found in close vicinity to GAD65 and D2Rs are expressed in dissociated rat cortical neurons *in vitro*

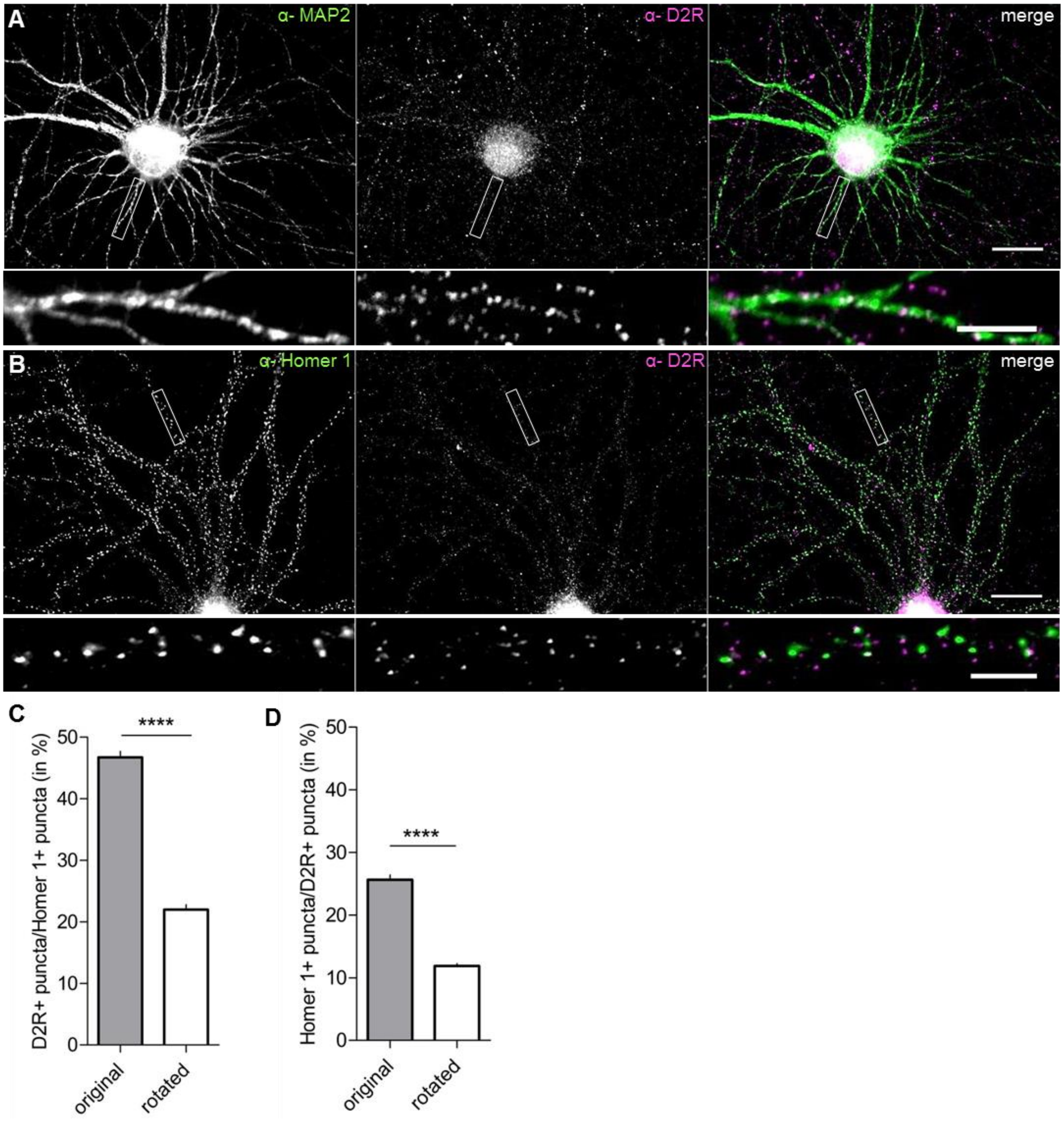

A) Rat dissociated cortical neurons (DIV21) were stained for the dendritic marker MAP2 (green) and D2R (magenta). D2Rs appear as bright puncta along dendrites (scale bar: 20 µm; close-up: 5 µm). **B)** D2Rs (magenta) are found in close vicinity of Homer 1-positive excitatory synapses (green) on rat dissociated cortical neurons (DIV21) (scale bar: 20 µm; close-up: 5 µm). **C)** Around 47 % of D2R-positive puncta are in close vicinity of Homer 1-positive synaptic puncta. The analysis of a 90° rotated image served as a quality control (original, 46.75 ± 0.9586, n = 8; rotated, 21.99 ± 0.8152, n = 8; average % ± SEM %; Paired t test; **** $P < 0.0001$). **D)** Around 25 % of Homer 1-positive synaptic puncta are in close vicinity of D2R-positive puncta. The analysis of a 90° rotated image served as a quality control (original, 25.66 ± 0.7576, n = 8; rotated, 11.87 ± 0.3838, n = 6; average % ± SEM %; Paired t test; **** $P < 0.0001$).

Supplementary Figure S4: Validation of knockdown efficiency of shRNA constructs shA4 and shA5

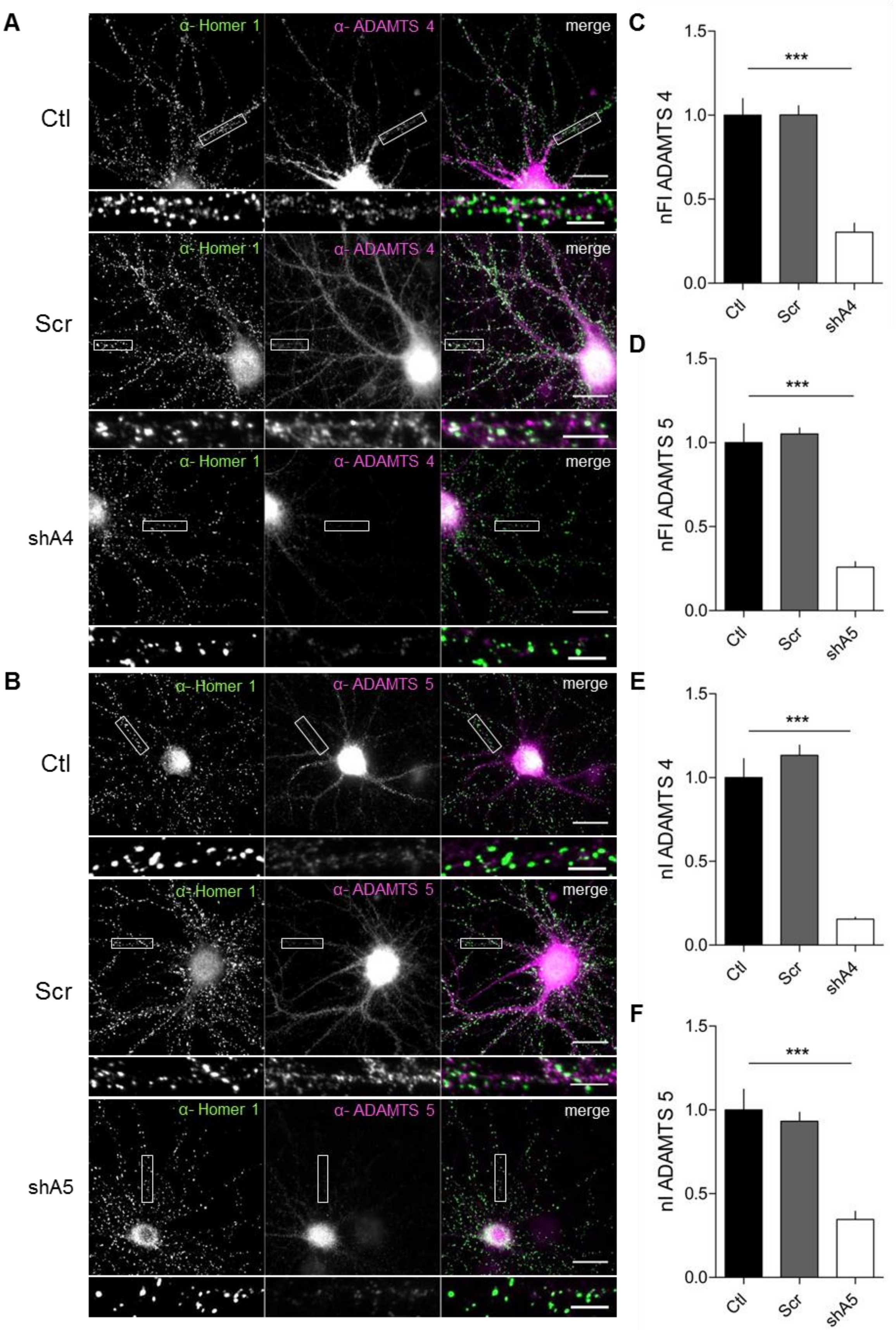

A) Rat dissociated cortical cultures (DIV14) were either non-infected (Ctl) or infected with scramble (Scr) or shADAMTS 4.2 (shA4). At DIV21 cultures were stained for the synaptic marker Homer 1 (green) and ADAMTS 4 (magenta) (scale bar: 20 µm; close-up: 5 µm). **B)** Rat dissociated cortical cultures (DIV14) were either non-infected (Ctl) or infected with Scr or shADAMTS 5.2 (shA5). At DIV21 cultures were stained for the synaptic marker Homer 1 (green) and ADAMTS 5 (magenta) (scale bar: 20 µm; close-up: 5 µm). **C)** Validation of knockdown efficiency at Homer 1-positive synapses revealed that construct shA4.2 shows an efficient knockdown (Ctl, 1 ± 0.0995, n = 5; Scr, 1.002 ± 0.0546, n = 5; shA4, 0.3034 ± 0.0543, n = 6; average ± SEM; One-way ANOVA; P = 0.001; Dunnett's Multiple Comparison Test; *** P<0.001). **D)** Also, at Homer 1-positive synapses construct shA5.2 displays an efficient knockdown for ADAMTS 5 (Ctl, 1 ± 0.1133, n = 5; Scr, 1.052 ± 0.0352, n = 4; shA5, 0.2588 ± 0.0326, n = 4; average ± SEM; One-way ANOVA; P < 0.001; Dunnett's Multiple Comparison Test; *** P<0.001). **E,F)** Knockdown efficiency was proved using western blot analysis (**E:** Ctl, 1 ± 0.113, n = 5; Scr, 0.9759 ± 0.0246, n = 5; shA4, 0.2461 ± 0.0188, n = 3; average ± SEM; One-way ANOVA; P = 0.001; Dunnett's Multiple Comparison Test; *** P<0.001; **F:** Ctl, 1 ± 0.1234, n = 6; Scr, 0.9407 ± 0.0379, n = 6; shA5, 0.3465 ± 0.0487, n = 4; average ± SEM; One-way ANOVA; P = 0.0003; Dunnett's Multiple Comparison Test; *** P<0.001) (nFI = normalized fluorescent intensity; nI = normalized intensity)

Supplementary Figure S5: ADAMTS 4 is expressed by neurons and astrocytes *in vitro*

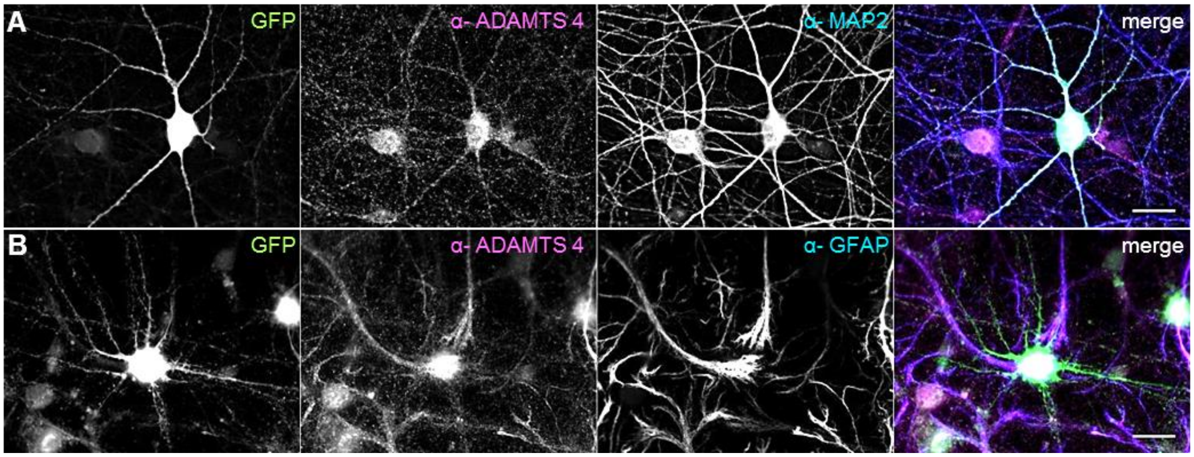

A,B) Rat dissociated cortical cultures (DIV14) were infected using shADAMTS4.1-AAV-GFP. At DIV 21 cells were stained for ADAMTS 4 (magenta) and the somato-dendritic marker MAP2 (A,blue) or the astrocytic marker GFAP (B, blue). The strong GFP signal (green) indicates the successful infection with the used AAV. Astrocytes display no GFP signal suggesting no infection with the used AAV (scale bar: 20 µm).

Supplementary Figure S3: Consistent correlation between the local BC cleavage and expression of postsynaptic protein Homer 1

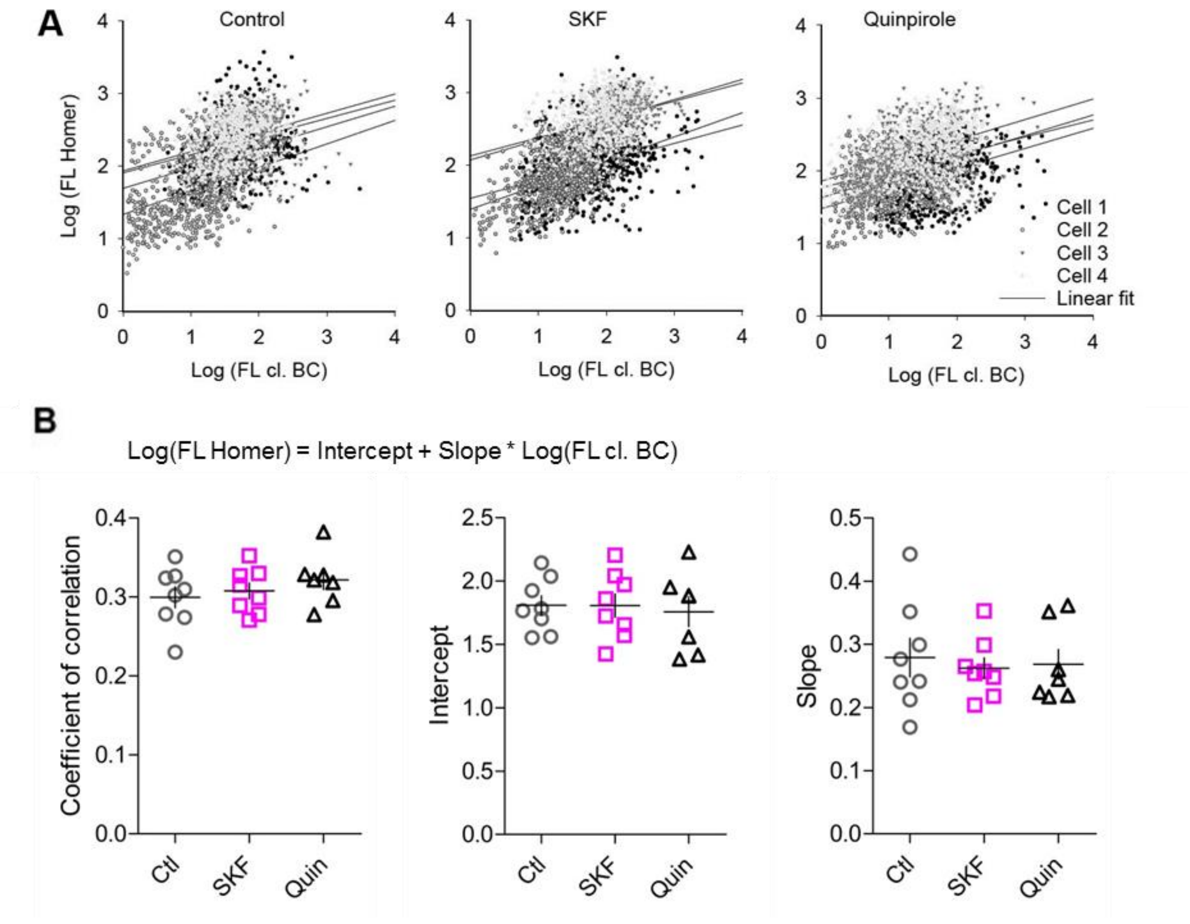

A) Values of log(FL Homer) and log(FL cl. BC) at individual synapses in four cells (one cell per culture preparation) in control conditions and after stimulation with SKF81297 or quinpirole. Linear regressions optimally fitting the relationship between the studied parameters are shown for all cells. B) Mean+SEM values of the Pearson coefficient of correlation, intercept and slope of linear regressions in n control conditions (n=8 coverslips) and after stimulation with SKF81297 (n=8) or quinpirole (n=7). There is no effect of treatment on any of these parameters (P>0.1, one-way ANOVA). The coefficients of correlations are significantly different from 0 (P<0.000001, one-sample t-test).

Supplementary Figure S6: Schematic illustration of the molecular mechanisms resulting in ECM remodeling after D1-like DA receptor activation

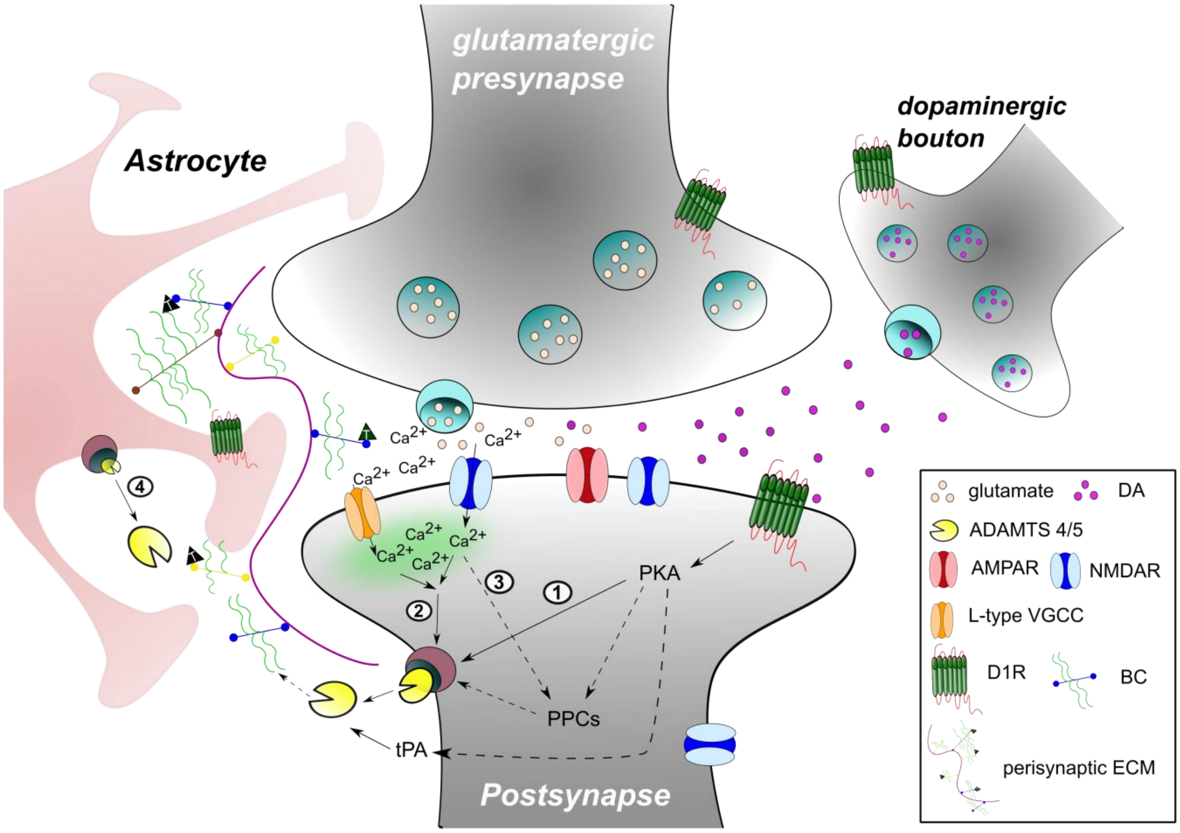

Activation of D1 DA receptors results in increased intracellular cAMP levels leading to downstream activation of PKA. Further, active PKA might be able to release already active proteases, such as ADAMTS-4 and ADAMTS-5 in the extracellular space to remodel the perisynaptic ECM (①). Besides its downstream signaling, stimulation of D1-like DA receptor can influence the Ca^{2+} influx through NMDARs and L-type VGCC. Enhanced Ca^{2+} influx activates CaMKII that might lead to the release of ADAMTS-4 and -5 into the extracellular space (②). D1-like DA receptor activation might result in a co-signaling of pathways ① and ②. All ADAMTS enzymes carry a pro-domain to keep them in an inactive state. After D1-like DA receptor stimulation, PKA activation and enhanced Ca^{2+} influx could result in the activation of pro-protein convertases (PPCs). Active PPCs themselves cleave off the pro-domain of ADAMTS-4 and -5 resulting in active enzymes ready for release (③). Beside PPCs tPA could be also extracellularly active upon D1-like receptor stimulation and could function as an activator for ADAMTS-4/-5. ADAMTS-4 and ADAMTS-5 are expressed also by astrocytes. Therefore, activation of neuronal or astrocytic D1-like DA receptors might cause a release of these enzymes by astrocytes as well (④).
